# Safety and Effectiveness of Ustekinumab for Crohn’s Disease With Perianal Manifestations: Ad hoc Analysis Data From 1-Year Post-Marketing Surveillance Study in Japan

**DOI:** 10.1093/crocol/otae035

**Published:** 2024-05-16

**Authors:** Katsumasa Nagano, Hiroaki Tsuchiya, Teita Asano, Hiroshi Yamazaki, Sonoko Tominaga, Takayuki Matsumoto

**Affiliations:** Janssen Pharmaceutical K.K., Tokyo, Japan; Janssen Pharmaceutical K.K., Tokyo, Japan; Janssen Pharmaceutical K.K., Tokyo, Japan; Janssen Pharmaceutical K.K., Tokyo, Japan; Janssen Pharmaceutical K.K., Tokyo, Japan; Division of Gastroenterology, Department of Internal Medicine, School of Medicine, Iwate Medical University, Japan

**Keywords:** Ustekinumab, Crohn’s disease, perianal manifestations, safety, effectiveness, Japan

## Abstract

**Background:**

Crohn’s disease (CD) is an immune-mediated inflammatory disorder of the gastrointestinal tract with perianal disease being one of the challenging possible manifestations. Here, we report, an ad hoc analysis of the safety and effectiveness of 1-year use of ustekinumab (UST) for CD in patients with perianal manifestations using post-marketing surveillance (PMS) data in Japan.

**Methods:**

Among 341 patients enrolled in the PMS, 229 and 224 patients who had baseline Crohn’s Disease Activity Index (CDAI) data used for evaluating perianal manifestations were included in the safety and efficacy analysis sets, respectively. Incidence of adverse drug reactions, clinical remission, the mean or its change in CDAI scores, and CDAI items were evaluated through week 52 in the presence or absence of perianal manifestations at baseline. The prevalence of perianal manifestations was also described.

**Results:**

Comparing patients with and without baseline perianal manifestations at week 52, there was no difference in ADR incidence (9.1% [*n* = 66] vs. 15.3% [*n* = 163]), no difference in clinical remission (68.3% vs. 59.9%; *P* = 0.269), and decreased mean change of CDAI score (−82.9 [*n* = 60] vs. −68.8 [*n* = 137]). The proportion of patients with perianal manifestations decreased after UST treatment in both biologics-naïve patients (23.5% [*n* = 4/17]) and patients who had received biologics (35.0% [*n* = 14/40]) at week 52.

**Conclusions:**

In Japanese clinical practice, UST is safe and effective in CD patients with and without perianal manifestations. The therapy might be also beneficial in those with manifestations regardless of prior use of other biologics.

## Introduction

Crohn’s disease (CD) is an immune-mediated inflammatory disease of the gastrointestinal tract.^1^ Perianal disease is one of the most challenging phenotypes of CD, and perianal fistula is an especially aggressive and debilitating manifestation of CD.^2,3^ It also includes anal fissure and perianal abscesses.^4^ The presence of perianal disease is recognized as a marker and predictor of the severe disabling disease course of CD.^4,5^ This can cause pain, swelling, and discharge in the perianal region and have a negative impact on healthcare-related quality of life.^4^ Recent data have demonstrated that fatigue and impairment of daily activities are greater in patients with perianal lesions than in patients without perianal lesions.^2^ Perianal manifestations in CD are present in up to a quarter of patients worldwide, in almost half of patients in Asia, including Japan, and the incidence of these manifestations is observed to increase with CD duration.^6,7^ Recent epidemiological studies have suggested a rapidly increasing incidence of CD in Japan.^8–10^

Perianal manifestations represent a significant therapeutic challenge.^11^ The current guidelines suggest a multidisciplinary approach consisting of a combination of noninvasive seton placement, stem cell therapy, and medical therapies including short-term antibiotics and long-term immunomodulators or biologic therapies.^12,13^ However, conventional medical therapy for the management of perianal manifestations in CD has its limitations. Steroids and aminosalicylates do not have any efficacy in perianal fistulizing CD and are not indicated in treatment algorithms.^14^ The use of antibiotics often shows symptomatic improvement but is associated with adverse events, such as diarrhea and poor efficacy. For biological therapies, the guideline recommends infliximab (IFX) as the first choice, and adalimumab in patients refractory or intolerant to IFX, for medical treatment of perianal disease, especially perianal fistula, while evidence supporting the use of the non-tumor necrosis factor (TNF) inhibitors, ustekinumab (UST), and vedolizumab (VED) remains too limited.^15^

UST is a fully-humanized monoclonal antibody, which binds to the p40 subunit common to both IL-12 and IL-23 and is approved for use in moderate-to-severe CD patients.^16,17^ A post hoc pooled analysis of data from randomized clinical trials recently demonstrated the efficacy of UST in inducing fistula response and remission.^18^ In addition, studies have suggested that UST is a potentially effective therapeutic option in perianal refractory CD.^19,20^ However, there is limited real-world evidence on the use of UST in patients with perianal disease, especially in Japan where perianal manifestations are common.^21^ Moreover, recent studies have reported that perianal disease is a predictive factor negatively impacting the effectiveness of UST.^22^ However, differences in the effect of UST on disease activity, including luminal disease activity, in patients with and without perianal manifestations have not yet been examined.

Recently, our post-marketing surveillance (PMS) has reported the safety and effectiveness of UST treatment for CD in Japanese patients.^23^ In this ad hoc analysis, we investigated the safety and effectiveness of the 1-year treatment with UST for CD in patients with and without perianal manifestations. Prevalence of perianal manifestations in CD patients was also investigated.

## Methods

This is an ad hoc analysis of data from a prospective, observational, and multicentre PMS conducted in Japan between May 2017 and December 2021. A detailed methodology of this study has been published.^23^ In brief, eligible patients were those with moderate-to-severe active CD who had failed or were intolerant to earlier treatment and were administered UST intravenous (IV) infusion based on the patient’s weight (~6 mg/kg), and subsequently administered 90 mg of UST subcutaneously at week 8, and according to the package insert, 90 mg every 8 or 12 weeks thereafter. Patients with a history of the use of UST were excluded. Some patients in clinical remission who had Crohn’s Disease Activity Index (CDAI) scores < 150 at baseline were enrolled in the PMS study. The observation period extended from the first dosing date of UST IV infusion to week 52 or the date of treatment completion/discontinuation. Among patients enrolled in the PMS, CD patients who had an evaluation of perianal manifestations at baseline were included in this ad hoc analysis. The evaluation was judged by the presence of perianal manifestations (anal fissure, fistula, or perianal abscess) or no symptoms as scored by CDAI. Patients with no record or no evaluation of CDAI items were excluded from this study. In clinical practice in Japan, perianal manifestations are generally evaluated by physicians based on perianal symptoms. If patients with CD are suspected of having perianal manifestations, physicians will refer them to proctologists as appropriate for further assessment and diagnostic tests, such as MRI or CT and examination under anesthesia.

### Safety

Safety evaluations included incidence of adverse drug reactions (ADRs) and serious ADRs (SADRs). The frequencies of patients with ADRs and incidence rates were reported by severity and preferred term (PT, MedDRA version [24.1]).

### Clinical Efficacy Evaluations

Efficacy evaluations were conducted in patients who had CDAI scores recorded at baseline and at least once during the study period. Effectiveness of UST from baseline to week 52 was evaluated by change of CDAI score from baseline (overall, with or without perianal manifestations); clinical remission (defined as CDAI < 150) at week 52 in the patients with or without perianal manifestations; prevalence of perianal manifestations overall, prevalence in each of 2 groups (biologic-naïve and -experienced), and prevalence in each of 2 subpopulations (patients with CDAI score ≥ 150 and patients with CDAI score < 150 at baseline). Changes in the CDAI subscores of general well-being (GWB), stool frequency (SF), and abdominal pain (AP) were also evaluated in patients with or without perianal manifestations after UST treatment. Using available data, changes in serum C-reactive protein (CRP) levels from baseline to week 52 were measured overall and in each group (with or without perianal manifestations).

#### Ethical considerations

The study was carried out in accordance with ethical principles outlined in the Declaration of Helsinki, the Japanese authorized standards for PMS, and Good Post-marketing Study Practice without intervening in the dosage and administration of UST. Good Post-marketing Study Practice does not require the patient’s consent to participate and the institutional review board’s approval of the study protocol at each medical facility; hence patients’ consent was not obtained.

#### Statistical analyses

Summary statistics of demographic and baseline characteristics were calculated for mean, standard deviation (SD), and median for continuous variables, and frequency and proportion for categorical variables. The present study used an as-observed case approach to handling all missing data except treatment discontinuation data. CDAI score was used for observed case analysis. The last observation carried forward (LOCF) method was applied to missing items of the CDAI in calculating CDAI scores, only in patients who had more than 4 of the 8 measured CDAI items. The summary statistics and 95% confidence interval (CI) for CDAI, CDAI sub-score, and CRP values, and the change from baseline at each visit were calculated. The proportions with 95% CIs of patients who achieved clinical remission at week 52 were tabulated. The *p*-value for the difference in clinical remission rate between patients with and without perianal manifestations at baseline was calculated by the Fisher exact test and used for descriptive or exploratory purposes.

## Results

### Study Population

**Figure 1.**
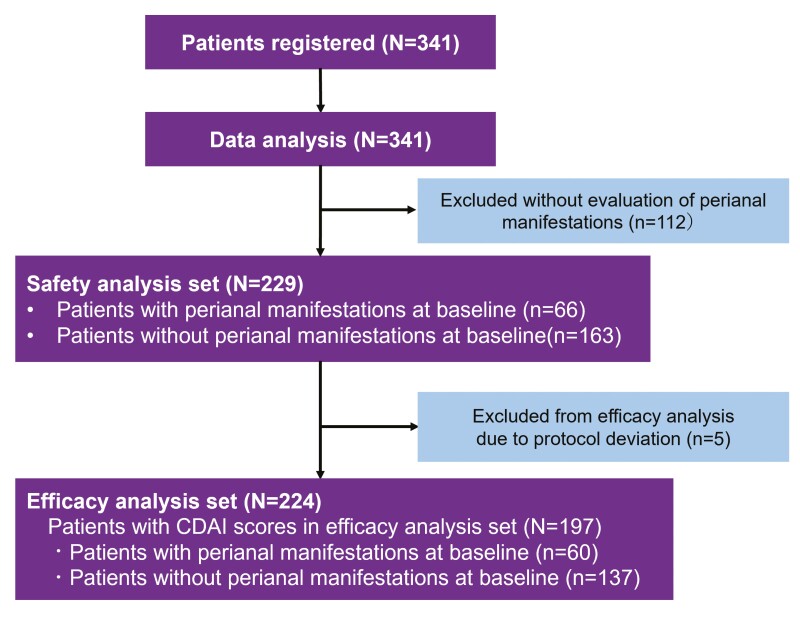
Patient flow diagram. CDAI, Crohn’s disease activity index.

The PMS study was conducted in 341 Japanese patients from 91 medical facilities in Japan.^23^ Patients who had been prescribed UST between May 2017 and April 2019 were enrolled. In this ad hoc analysis using CDAI data, the safety and effectiveness of UST for perianal manifestations in CD patients were assessed. After 112 patients without evaluation of perianal manifestations at baseline were excluded, 229 patients (66 patients with perianal manifestations at baseline and 163 patients without perianal manifestations at baseline) were included in the safety analysis set (Figure 1), and 224 patients were included in the efficacy analysis set (full analysis set). Of these, 197 patients had CDAI scores (60 patients with perianal manifestations and 137 patients without perianal manifestations) and were evaluated. Although 11 patients showed the emergence of perianal manifestations after UST treatment, all of them continued UST treatment for 52 weeks (Supplementary Table S1).

In the safety analysis set, there was a higher proportion of male (153/229 [66.8%]) than female (76/229 [33.2%]) patients (Table 1). The median age was 38.0 years, and the mean (SD) duration of CD at baseline was 11.4 (9.4) years. Most of the patients had ileocolonic (159/229 [69.4%]) and non-structuring and non-penetrating CD (116/229 [50.7%]). Of the 229 patients, 52/229 (22.7%) had extraintestinal manifestation (EIM), and a majority of the patients (200/229 [87.3%]) had CDAI scores at baseline. Between patients with and without perianal manifestations, patient characteristics and demographics were generally comparable. In patients without versus patients with perianal manifestations, little difference was observed in disease duration (12.4 ± 9.9 vs. 9.0 ± 7.6 years), colonic disease location (8.6% vs. 18.2%), EIM (14.7% vs. 42.4%), and prior use of ≥ 2 biologics (96.8% vs. 85.1%).

**Table 1.** Demographics and baseline characteristics.

Factor			Number of patients, %					
			Safety analysis set		Without perianal disease		With perianal disease	
Number of analysis set			229		163		66	
Sex	Male		153	(66.8)	109	(66.9)	44	(66.7)
	Female		76	(33.2)	54	(33.1)	22	(33.3)
Age	Mean ± Standard deviation		37.2 ± 13.6		38.7 ± 14.2		33.3 ± 11.1	
BMI (kg/m2)	Mean ± Standard deviation		20.4 ± 3.6		20.3 ± 3.6		20.7 ± 3.7	
Disease duration (years)	Mean ± Standard deviation		11.4 ± 9.4		12.4 ± 9.9		9.0 ± 7.6	
	Unknown		15	(6.6)	10	(6.1)	5	(7.6)
Affected areas	Disease location	Ileal	47	(20.5)	39	(23.9)	8	(12.1)
(There is duplication)		Ileocolonic	159	(69.4)	113	(69.3)	46	(69.7)
		Colonic	26	(11.4)	14	(8.6)	12	(18.2)
	Disease behavior	Non-stricturing, non-penetrating	116	(50.7)	80	(49.1)	36	(54.5)
		Penetrating	51	(22.3)	33	(20.2)	18	(27.3)
		Stricturing	112	(48.9)	81	(49.7)	31	(47.0)
Smoking history	Yes		43	(18.8)	34	(20.9)	9	(13.6)
	Current smoker		22	(51.2)	17	(50.0)	5	(55.6)
	Past smoker		21	(48.8)	17	(50.0)	4	(44.4)
	Unknown		19	(8.3)	13	(8.0)	6	(9.1)
Comorbidities	Yes		88	(38.4)	65	(39.9)	23	(34.8)
EIM	Yes		52	(22.7)	24	(14.7)	28	(42.4)
Surgical history	Yes		111	(48.5)	83	(50.9)	28	(42.4)
Prior use of biologics	No		56	(24.5)	37	(22.7)	19	(28.8)
	Yes		173	(75.5)	126	(77.3)	47	(71.2)
By number of drugs	1		11	(6.4)	4	(3.2)	7	(14.9)
	≥2		162	(93.6)	122	(96.8)	40	(85.1)
Type of biologics used	Infliximab		134	(77.5)	99	(78.6)	35	(74.5)
(There is duplication)	Adalimumab		91	(52.6)	66	(52.4)	25	(53.2)
	Vedolizumab		1	(0.6)	0	(0.0)	1	(2.1)
Prior use of other non-biologic treatment	Yes		202	(88.2)	147	(90.2)	55	(83.3)
(There is duplication)	Steroid		88	(43.6)	62	(42.2)	26	(47.3)
	AZA		53	(26.2)	39	(26.5)	14	(25.5)
	6-MP		13	(6.4)	12	(8.2)	1	(1.8)
	Methotrexate		2	(1.0)	2	(1.4)	0	(0.0)
	5-ASA		163	(80.7)	118	(80.3)	45	(81.8)
	Antibiotics		18	(8.9)	10	(6.8)	8	(14.5)
	Enteral nutrition		24	(11.9)	21	(14.3)	3	(5.5)
Concomitant CD medication at baseline	Yes		193	(84.3)	140	(85.9)	53	(80.3)
(There is duplication)	Steroid		72	(37.3)	47	(33.6)	25	(47.2)
	AZA		49	(25.4)	36	(25.7)	13	(24.5)
	6-MP		12	(6.2)	10	(7.1)	2	(3.8)
	Methotrexate		2	(1.0)	2	(1.4)	0	(0.0)
	5-ASA		158	(81.9)	113	(80.7)	45	(84.9)
	Antibiotics		7	(3.6)	5	(3.6)	2	(3.8)
	Enteral nutrition		23	(11.9)	19	(13.6)	4	(7.5)
Baseline CDAI score	No		29	(12.7)	23	(14.1)	6	(9.1)
	Yes		200	(87.3)	140	(85.9)	60	(90.9)
	< 150		69	(34.5)	53	(37.9)	16	(24.2)
	≥150 and < 220		56	(28.0)	38	(27.1)	18	(27.3)
	≥220 and < 450		72	(36.0)	46	(32.9)	26	(39.7)
	≥450		3	(1.5)	3	(2.1)	0	(0.0)

AZA, azathioprine; CD, Crohn’s disease; CDAI, Crohn’s disease activity index; EIM, extraintestinal manifestations; 6-MP, 6-mercaptopurine.

### Safety Findings

At week 52, the overall incidence of ADRs and SADRs was 13.5% (*n* = 31/229) and 8.3% (*n* = 19/229), respectively (Table 2). The incidence of ADRs by PT was highest for worsening of CD (2.6% [*n* = 6/229]), followed by pyrexia (1.7% [*n* = 4/229]), anal abscess (1.3% [*n* = 3/229]), and upper respiratory tract inflammation (1.3% [*n* = 3/229]). The most frequently reported SADR was worsening CD (2.6% [*n* = 6/229]). There was no difference in ADR incidence between patients with (9.1% [*n* = 6/66]) and without perianal manifestations (15.3% [*n* = 25/163]) while the incidence rate of ADRs in each group was too low for detailed comparison.

**Table 2.** Incidence of adverse drug reactions with or without perianal manifestations at baseline (safety analysis set).

Safety analysis set	With perianal manifestations (*N* = 66)			Without perianal manifestations (*N* = 163)			All (*N* = 229)		
	SADR	Non-SADR	ADR	SADR	Non-SADR	ADR	SADR	Non-SADR	ADR
Number of patients	3	3	6	16	9	25	19	12	31
Number of events	4	3	7	18	21	39	22	24	46
Incidence rate (%)	4.5	4.5	9.1	9.8	5.5	15.3	8.3	5.2	13.5
*ADRs reported in > 0.5% of patients, n (%)*									
Worsening of CD	1 (1.5)	0 (0.0)	1 (1.5)	5 (3.1)	0 (0.0)	5 (3.1)	6 (2.6)	0 (0.0)	6 (2.6)
Anal abscess	0 (0.0)	0 (0.0)	0 (0.0)	3 (1.8)	0 (0.0)	3 (1.8)	3 (1.3)	0 (0.0)	3 (1.3)
Upper respiratory tract inflammation	0 (0.0)	0 (0.0)	0 (0.0)	0 (0.0)	3 (1.8)	3 (1.8)	0 (0.0)	3 (1.3)	3 (1.3)
Pyrexia	0 (0.0)	1 (1.5)	1 (1.5)	0 (0.0)	3 (1.8)	3 (1.8)	0 (0.0)	4 (1.7)	4 (1.7)
Influenza	0 (0.0)	0 (0.0)	0 (0.0)	0 (0.0)	2 (1.2)	2 (1.2)	0 (0.0)	2 (0.9)	2 (0.9)
Intestinal obstruction	1 (1.5)	0 (0.0)	1 (1.5)	1 (0.6)	0 (0.0)	1 (0.6)	2 (0.9)	0 (0.0)	2 (0.9)

ADR, adverse drug reaction; CD, Crohn’s disease; SADR, serious adverse drug reaction.

MedDRA version (24.1).

### Effectiveness Findings

#### The changes in CDAI scores

**Figure 2.**
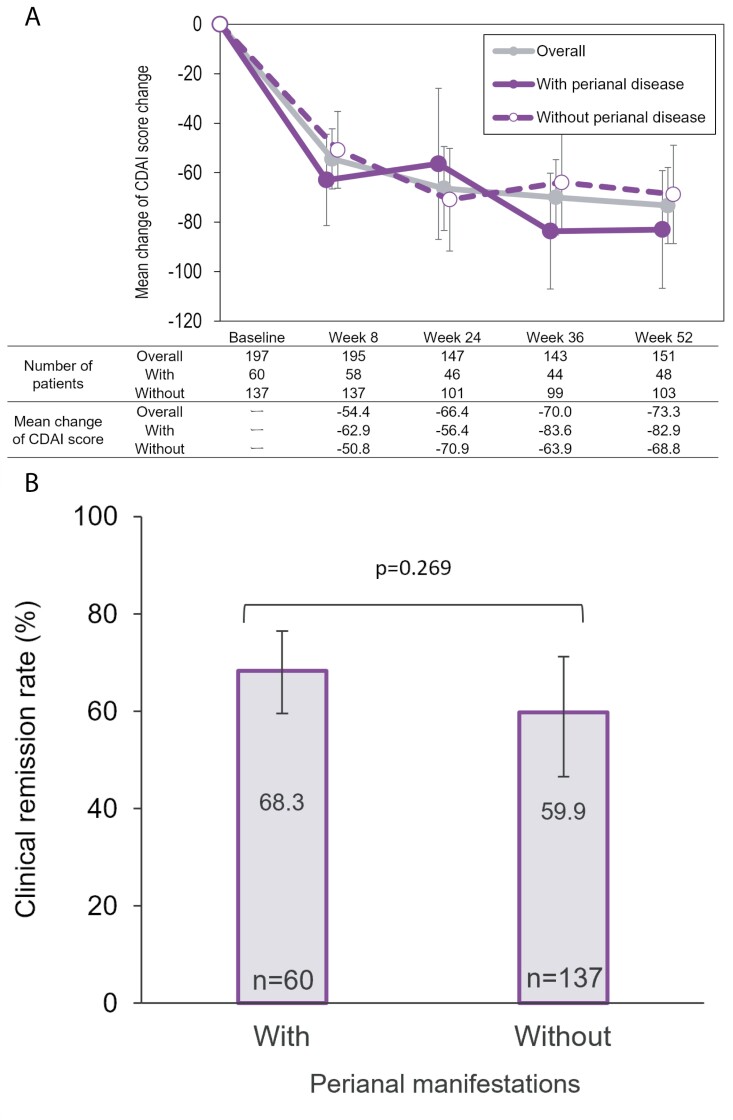
The mean change of CDAI scores and clinical remission by the presence or absence of perianal manifestations. (A) Mean change of CDAI score from baseline (overall, with or without perianal manifestations at baseline). (B) Clinical remission rate at week 52 (with or without perianal manifestations at baseline). The *P*-value was calculated by Fisher’s exact test. Error bar shows 95% confidence intervals. CDAI, Crohn’s Disease Activity Index.

The mean (95% CI) change in CDAI score decreased gradually in a time-dependent manner through week 52 regardless of existing perianal manifestations (Figure 2A). It was slightly higher in patients who had perianal manifestations at baseline (*n* = 48, −82.9 [−106.7 to −59.2]) at week 52, as compared to those without (*n* = 103, −68.8 [−88.7 to −48.9]). Patients with CDAI score ≥ 150 at baseline with perianal manifestations achieved greater reduction as compared to patients without perianal manifestations (*n* = 44, 234.5 [214.5 to 254.6] to *n* = 35, 118.5 [90.9 to 146.1] vs. *n* = 85, 253.6 [233.0 to 274.3] to *n* = 65, 156.8 [134.1 to 179.4] at week 52, Supplementary Figure S1a). While in patients with and without perianal manifestations who had baseline CDAI score < 150, the reduction was similar (*n* = 16, 106.7 [87.7 to 125.8] to *n* = 13, 91.1 [46.4 to 135.7] vs. *n* = 52, 84.6 [74.7 to 94.4] to *n* = 38, 70.3 [52.4 to 88.2] at week 52, Supplementary Figure S1B). The rates of clinical remission were similar between patients with perianal manifestations (*n* = 60, 68.3% [55.0% to 79.7%]) and patients without (*n* = 137, 59.9% [51.1% to 68.1%]; *p* = .269, Figure 2B).

### SF, AP, and GWB

The overall SF scores decreased through 52 weeks (Supplementary Figure S2A). The decrease in SF score from baseline was slightly greater in patients with perianal manifestations (*n* = 60, 23.3 [19.1 to 27.5]) to week 52 (*n* = 48, 13.4 [9.3 to 17.4]) as compared to patients without perianal manifestations (baseline [*n* = 137]: 25.0 [21.1 to 29.0] to week 52 [*n* = 103]: 18.4 [14.6 to 22.1]). Overall, no difference in AP score and GWB score was observed from baseline through 52 weeks in both patients with (AP, baseline [*n* = 60]: 4.10 [3.1 to 5.1] to week 52 [*n* = 48]: 1.98 [1.0 to 2.9], GWB, baseline [*n* = 60]: 7.03 [5.8 to 8.3] to week 52 [*n* = 48]: 2.81 [1.6 to 4.0]) and without perianal manifestations at baseline (AP, baseline [*n* = 137]: 4.41 [3.5 to 5.3] to week 52 [*n* = 103]: 2.15 [1.4 to 2.9], GWB, baseline [*n* = 137]: 6.87 [5.8 to 7.9] to week 52 [*n* = 103]: 3.17 [2.4 to 4.0]; Supplementary Figure S2B and S2C).

### Prevalence of Perianal Manifestations

**Figure 3.**
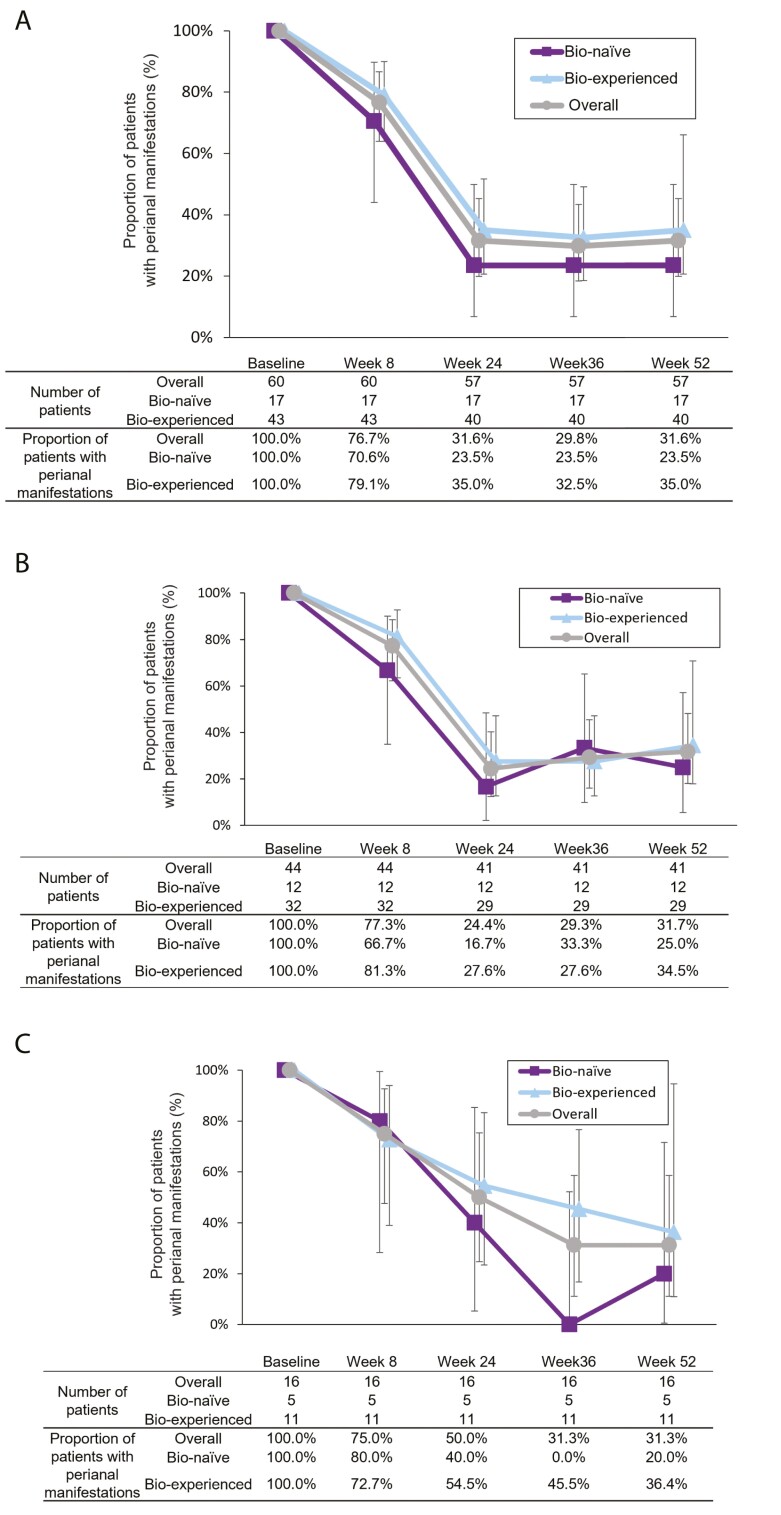
Proportion of patients with perianal manifestations. (A) Proportion of patients with perianal manifestations by prior biologic use (patients with perianal manifestations at baseline). (B) Proportion of patients with perianal manifestations by prior biologic use (patients with perianal manifestations and CDAI score ≥ 150 at baseline). (C) Proportion of patients with perianal manifestations by prior biologic use (patients with perianal manifestations and CDAI score < 150 at baseline). Error bar shows 95% confidence intervals. CDAI, Crohn’s disease activity index.

Of the 60 patients who had perianal manifestations at baseline, 43 were biologic experienced while 17 were biologic naïve. The proportion of patients with perianal manifestations decreased after UST treatment in both biologic-naïve patients (23.5% [*n* = 4/17]) and biologic-experienced patients (35.0% [*n* = 14/40]) at week 52 (Figure 3A). The reduction in the perianal manifestations rate from baseline to week 52 was similar between the subpopulation with CDAI scores ≥ 150 (31.7% [*n* = 13/41]; Figure 3B) and the subpopulation with CDAI scores < 150 (in clinical remission; 31.3% [*n* = 5/16]; Figure 3C).

Among patients with CDAI scores in the efficacy analysis set (*n* = 197), the perianal manifestations rate at baseline was slightly higher in biologic-naïve patients (37.8% [*n* = 17/45]) than in biologic-experienced patients (28.3% [*n* = 43/152]). The perianal manifestations rate at week 52 was 12.4% (*n* = 23/186) in the entire set, and similar between biologic-experienced patients (12.6% [*n* = 18/143]) and biologic-naïve patients (11.6% [*n* = 5/43]; Supplementary Figure S3).

### Serum CRP Level

A mean CRP was seen to decrease as early as week 8, continued to decrease until week 24, and remained steady until week 52 (Supplementary Figure S4). A greater decrease in mean CRP (95% CI) was observed in patients without perianal manifestations at baseline through week 24 (baseline [*n* = 136]: 1.45 [1.1 to 1.8] to week 24 [*n* = 113]: 0.72 [0.5 to 0.9]) as compared those with perianal manifestations (baseline [*n* = 60]: 1.25 [0.8 to 1.7] to week 24 [*n* = 50]: 0.81 [0.4 to 1.2]). However, the mean CRP continued to decrease from weeks 36 to 52 in patients with perianal manifestations at baseline (week 36 [*n* = 53]: 0.61 [0.3 to 0.9] to week 52 [*n* = 53]: 0.56 [0.3 to 0.8]) but not in those without perianal manifestations (week 36 [*n* = 116]: 0.94 [0.6 to 1.3] to week 52 [*n* = 115]: 1.04 [0.7 to 1.4]).

## Discussion

This is the first evidence from a PMS study to show the safety and effectiveness of 1-year treatment with UST for CD in Japanese patients with and without perianal manifestations. Although a beneficial effect of UST for perianal manifestations in CD patients has been suggested, the data published so far is from a limited number of patients, especially patients in Japan where the prevalence of the manifestation is higher. Moreover, the difference in efficacy between the patients with and without perianal manifestations remains unknown. However, the present study demonstrated similar effectiveness for UST in patients with or without perianal manifestations and no new safety concerns, indicating a therapeutic benefit of using UST regardless of existing perianal manifestations.

The prevalence of perianal manifestations decreased through week 52 regardless of prior biologic use. In the present PMS, there was no difference in ADR incidence rate between those with and without perianal manifestations, which indicates the presence of perianal manifestations at baseline does not affect the rate. The frequently reported ADRs (worsening of CD, pyrexia, perianal abscess, and upper respiratory tract inflammation) were similar to those observed in the main analysis of the same PMS conducted in 341 patients.^23^ The IM-UNITI trial conducted in patients with CD including patients with perianal fistulas reported no evidence of increased risk of opportunistic infections or tuberculosis (TB) in those receiving maintenance or long-term therapy. In addition, through 5 years of UST maintenance treatment, there was no increase in the risk of death, anaphylactic and delayed hypersensitivity (serum-like sickness) reactions, or malignancy.^24^ Similarly, the present study found no evidence of increased risk of opportunistic infections, TB, or death. Moreover, the ADRs reported in this PMS were in line with ADRs in the previously available safety profile.^21,23,25,26^

Results of this study showed gradual reduction in the mean and mean change of CDAI scores through week 52, indicating the effectiveness of UST in real-world clinical practice over 52 weeks both in CD patients with and without perianal manifestations. A similar trend was observed in serum CRP levels with a slightly greater reduction in patients without perianal manifestations. Additionally, there was no difference in clinical remission rate between patients with or without perianal manifestations. These findings indicate that UST may be considered a potential treatment option regardless of existing perianal manifestations. Since perianal manifestations, especially perianal fistulas, are an important prognostic factor for treating CD patients, physicians may generally prefer to use TNF inhibitors in clinical practice in accordance with ECCO guidelines.^15^ However, our finding suggests that not only TNF inhibitors, but also UST can be used to treat patients with perianal manifestations, and the presence of perianal manifestations may not be a factor influencing the choice of UST as treatment. As with CDAI scores, the subscores for SF, AP, and GWB were improved in patients with perianal manifestations at Week 52. While the decrease in mean SF score was slightly higher in patients with perianal manifestations, perianal manifestations had no impact on the reduction of mean AP and GWB scores in patients with CD.

As for the prevalence of perianal manifestations, our data showed a decrease through week 52 after UST treatment in both biologic-naïve and biologic-experienced patients, suggesting that UST is a beneficial treatment for perianal manifestations, regardless of a prior use of biologics. However, since the number of these patients was small, the result should be interpreted cautiously. Also, we observed a similar decrease in the proportion of patients with perianal manifestations at week 52 in both subpopulations (baseline CDAI scores ≥ 150, not in remission, baseline CDAI < 150, in remission). This may suggest the usefulness of UST treatment in CD patients with perianal manifestations, even if patients are in clinical remission. Real-world cohort studies have reported fistula response rates of up to 66% after 6 months of treatment.^27,28^ The rate of perianal manifestation disappearance at week 52 in the present study was similar at 68.4% (with the perianal disease rate being 31.6%, Figure 3A). Results from the SEAVUE and STARDUST trials have recently reported the resolution of half of active perianal fistulas after 1 year of maintenance treatment with UST.^29^ Furthermore, a systematic review with meta-analysis indicated that 41% of fistulizing perianal CD patients treated with UST experienced a fistula response, and the proportion increased to 56% after a year of therapy.^30^ The percentages in our study were similar to the ones in these reports. However, our survey evaluated efficacy based on the presence or absence of perianal manifestations, which is different from the criteria used in clinical trials (at least 50% decrease or closure of external fistula opening with drainage) and previously reported criteria used in real-world clinical practice (provider’s physical examination and without need for medical or surgical intervention). Therefore, our result should be interpreted cautiously. Moreover, our study lacked rigorous evaluation for perianal manifestations, such as proctological examinations and MRI/CT imaging tests. The ECCO guideline recommends anti-TNF agents based on results from a randomized, placebo-controlled, ACCENT 2 trial for IFX and post hoc analyses of the CHARM and CHOICE trials for ADA.^15^ The ENTERPRISE study has recently reported that VED improved perianal fistula identified by MRI.^31^ These studies demonstrated efficacy compared with placebo or evaluated efficacy by MRI/CT imaging. Thus, further randomized, placebo-controlled studies, such as the USTAP study are required to clarify UST effectiveness for perianal manifestations, especially fistula.^32^

Considering the presence of perianal manifestations is a positive predictor of poor prognosis for CD^4,5^ and a negative predictor for UST effectiveness,^22^ our findings should be important information to have when treating CD patients with perianal manifestations. Moreover, although the ACCENT 2 and CHARM trials showed anti-TNF agents are effective in perianal fistula treatment, we cannot ignore that there are primary non-responders to anti-TNF agents and patients who have to switch to other classes of biologics due to loss of response and development of severe AEs. Although the sample size in our study was small, the incidence of perianal manifestations was no different between biologic-naïve and -experienced patients. A recent report demonstrated that among patients who failed to respond to a first anti-TNF agent, VED use was associated with decreased perianal fistula closure.^33^ These findings may support the use of UST as a potential treatment option for perianal manifestations even in patients switching from an anti-TNF agent to another class of biologics.

There are several limitations in this study. Firstly, not all perianal manifestations in the present study were diagnosed and assessed by experts (surgeons and proctologists). In the present study, perianal manifestations were generally evaluated by physicians based on perianal symptoms and patients were referred to proctologists when necessary for further assessment and diagnostic tests. In addition, this study lacked evaluation by objective methods under anesthesia and using monitoring tools, such as pelvic MRI/CT, and lacked evaluation of other perianal manifestations, such as skin tags, hemorrhoids, and ulcers. Secondly, this is an ad hoc analysis of PMS results so there were missing values for CDAI, which were imputed by the use of an LOCF methodology. As-observed analysis was used for effectiveness analysis. The use of observed case analysis usually provides a better percentage of efficacy than intention-to-treat analysis. In addition, patients who had discontinued UST treatment during the 52 weeks and those who had no known assessment of perianal manifestations were not included in this study. Therefore, our results include several biases. Thirdly, no control group was included and comparisons were made between biologic-naïve and -experienced groups and with and without perianal manifestations groups. Additionally, our study is limited to generalizability and suffers from biases, such as selection bias due to the ad hoc prospective, observational nature of the study. The final limitation is that only patients who were treated in Japanese facilities were included.

## Conclusions

In clinical practice, UST had an acceptable safety profile and was effective in Japanese patients with CD regardless of the presence of perianal manifestations or prior biologics use. Therefore, it may have potential as a treatment for the manifestations regardless of prior use of other biologics and for CD regardless of the presence of the perianal manifestations and prior biologic use.

## Supplementary Material

otae035_suppl_Supplementary_Materials

## Data Availability

The data underlying this article cannot be shared publicly due to confidentiality clauses signed with participating medical institutions. Research data are not shared due to confidentiality clauses signed with medical facilities.

## Poster Presentation

2022 Digestive Disease Week^®^ (San Diego, CA, US), May 21–24, 2022,

The 10th Annual Meeting of Asian Organization for Crohn’s & Colitis (Virtual Congress), June 6–18, 2022,

## Oral Presentation

The 13th Japanese Society for Inflammatory Bowel Disease (Osaka, Japan), November 25–26, 2022.
