# Supplementary material for: Safety and Effectiveness of Ustekinumab for Crohn’s Disease With Perianal Manifestations: Ad hoc Analysis Data From 1-Year Post-Marketing Surveillance Study in Japan
Source: Crohns Colitis 360. 2024 May 16;6(2):otae035. doi: 10.1093/crocol/otae035 (PMC11143479; doi:10.1093/crocol/otae035)
Supplement: otae035_suppl_Supplementary_Materials [file otae035_suppl_supplementary_materials.docx]

# SUPPLEMENTAL MATERIAL

## Table S1 Incidence of perianal manifestations and continuation rate of ustekinumab at last observation for patients with perianal manifestations at all visits except the baseline visit (Effectiveness analysis set)

|  | Number of patients |  | |
| --- | --- | --- | --- |
| Effectiveness analysis set | 224 |  |  |
| ∟Patient with CDAI | 197 |  |  |
| ∟Patients without perianal manifestations at baseline | 137 | Presence of perianal manifestations at any visit other than baseline (%) | |
|  |  | 11 | (8.0%) |
| ∟Presence of perianal manifestations at any visit other than baseline | 11 | Patients with perianal manifestations who continued ustekinumab at last observation (%) | |
|  |  | 11 | (100.0%) |

CDAI, Crohn’s Disease Activity Index.

## Figure S1 Mean value in CDAI scores

| 1. Patients with CDAI ≥150 at baseline   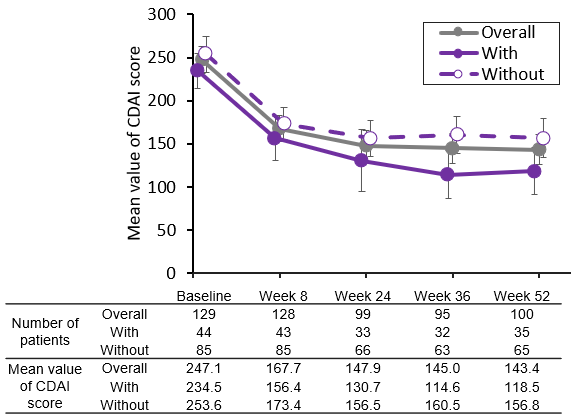  Error bar shows 95% confidence intervals.  CDAI, Crohn’s Disease Activity Index;  With, Patients with perianal manifestations at baseline;  Without, Patients without perianal manifestations at baseline.   1. Patients with CDAI<150 at baseline   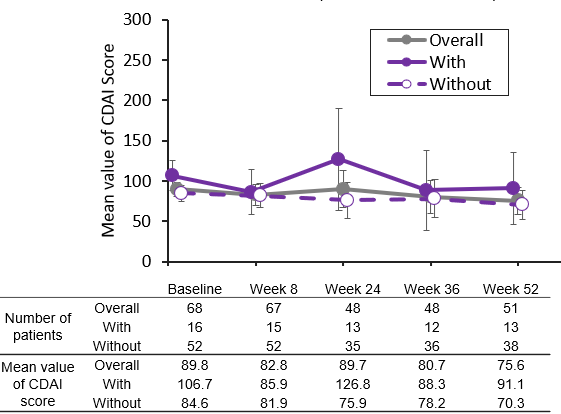  Error bar shows 95% confidence intervals.  CDAI, Crohn’s Disease Activity Index;  With, Patients with perianal manifestations at baseline;  Without, Patients without perianal manifestations at baseline. |
| --- |

## Figure S2 Efficacy of CDAI sub-score in patients with or without of perianal manifestations at baseline.

| 1. Mean value in stool frequency score   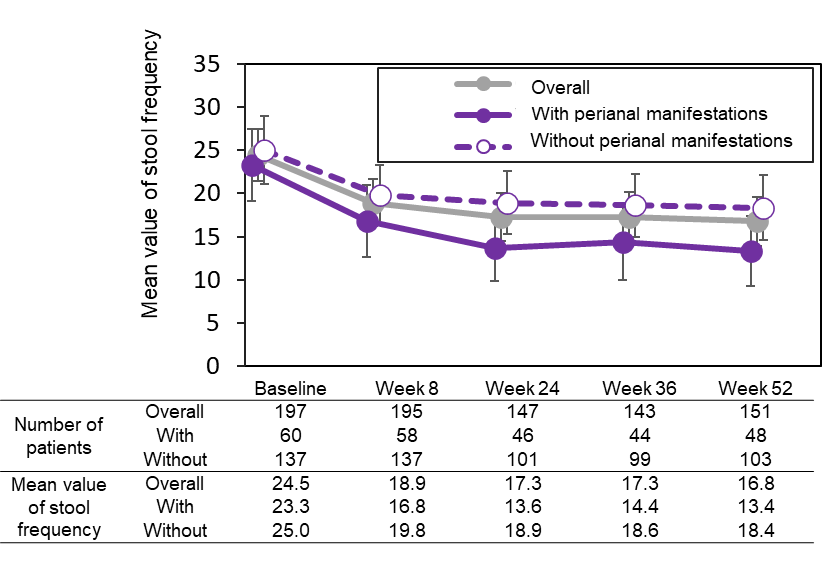  Error bar shows 95% confidence intervals.   1. Mean value of abdominal pain score   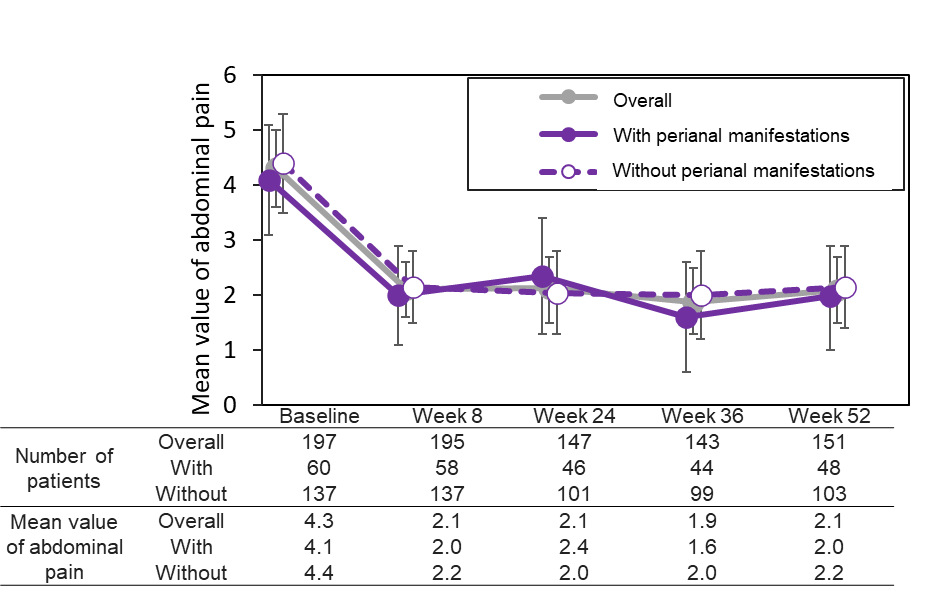  Error bar shows 95% confidence intervals.   1. Mean value of general well-being score   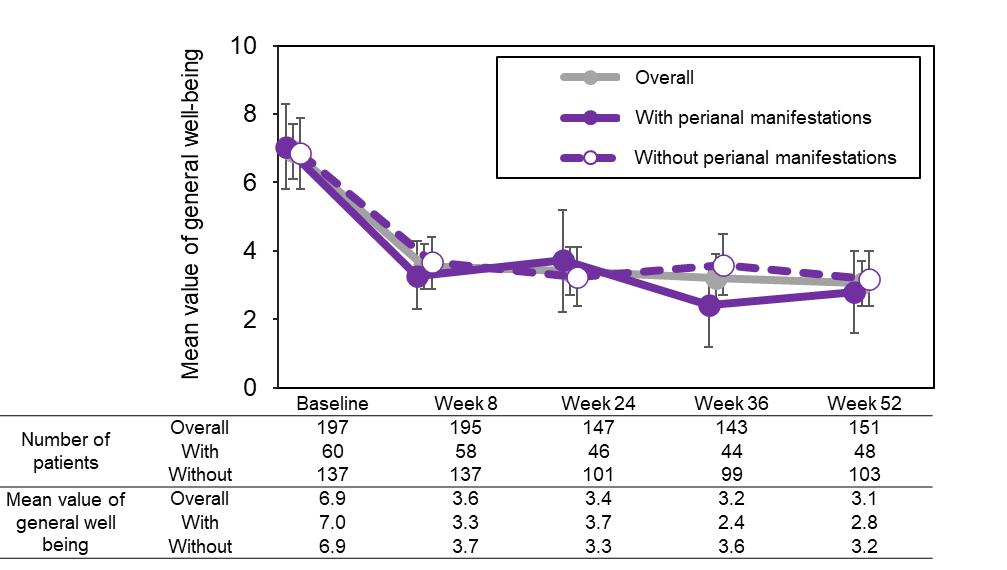  Error bar shows 95% confidence intervals. |
| --- |

## Figure S3 Changes in the perianal manifestations rates over time.

| 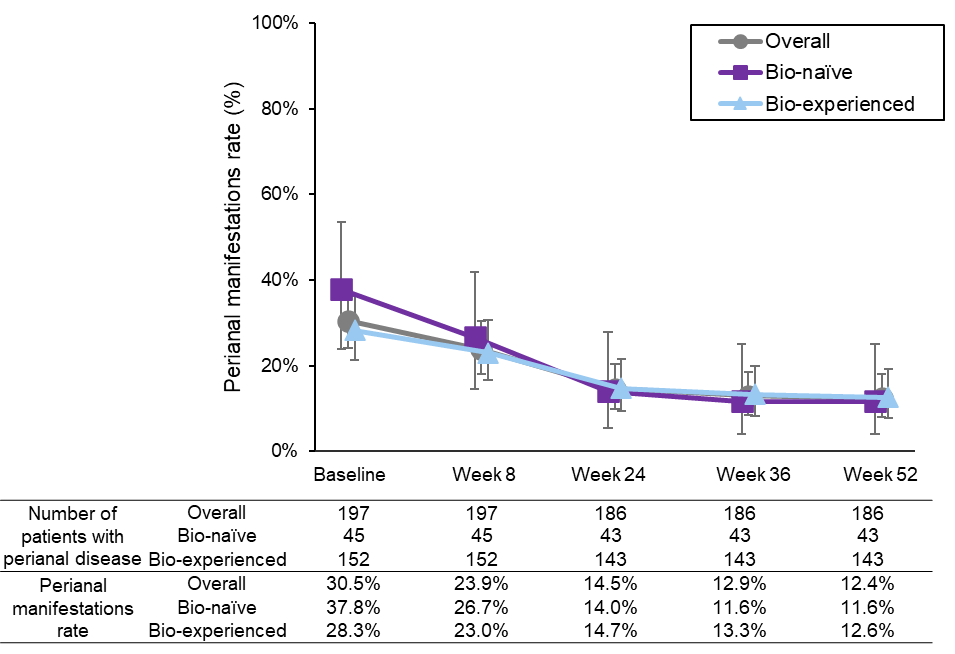  Error bar shows 95% confidence intervals. |
| --- |

## Figure S4 Mean change in CRP levels

| 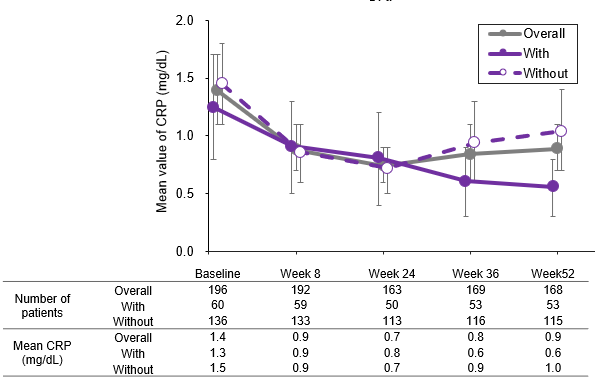  Error bar shows 95% confidence intervals.  CRP, C-reactive protein;  With, Patients with perianal manifestations at baseline;  Without, Patients without perianal manifestations at baseline. |
| --- |
